# Natural Compounds Rosmarinic Acid and Carvacrol Counteract Aluminium-Induced Oxidative Stress

**DOI:** 10.3390/molecules25081807

**Published:** 2020-04-15

**Authors:** Juste Baranauskaite, Ilona Sadauskiene, Arunas Liekis, Arturas Kasauskas, Robertas Lazauskas, Ugne Zlabiene, Ruta Masteikova, Dalia M. Kopustinskiene, Jurga Bernatoniene

**Affiliations:** 1 Department of Analytical and Toxicological Chemistry, Medical Academy, Lithuanian University of Health Sciences, LT-50161 Kaunas, Lithuania; Juste.Baranauskaite@lsmuni.lt; 2 Neuroscience Institute, Lithuanian University of Health Sciences, LT-50161 Kaunas, Lithuania; Ilona.Sadauskiene@lsmuni.lt (I.S.); Arunas.Liekis@lsmuni.lt (A.L.); 3 Department of Biochemistry, Medical Academy, Lithuanian University of Health Sciences, LT-50161 Kaunas, Lithuania; Arturas.Kasauskas@lsmuni.lt; 4 Institute of Physiology and Pharmacology, Medical Academy, Lithuanian University of Health Sciences, LT-50161 Kaunas, Lithuania; Robertas.Lazauskas@lsmuni.lt; 5 Institute of Pharmaceutical Technologies, Medical Academy, Lithuanian University of Health Sciences, LT-50161 Kaunas, Lithuania; Ugne.Cizauskaite@lsmuni.lt; 6 Department of Pharmaceutics, University of Veterinary and Pharmaceutical Sciences Brno, 61242 Brno, Czech Republic; masteikovar@vfu.cz; 7 Department of Drug Technology and Social Pharmacy, Medical Academy, Lithuanian University of Health Sciences, LT-50161 Kaunas, Lithuania

**Keywords:** aluminum toxicity, rosmarinic acid, carvacrol, oxidative stress, lipid peroxidation, brain, liver

## Abstract

Aluminum accumulation, glutathione (GSH) and malondialdehyde (MDA) concentrations as well as catalase (CAT) and superoxide dismutase (SOD) activities were determined in erythrocytes and brain and liver homogenates of BALB/c mice treated with Al^3+^ (7.5 mg/kg/day (0.15 LD_50_) as AlCl_3_ (37.08 mg/kg/day), whereas HCl (30.41 mg/kg/day) was used as Cl^−^ control, the treatments were performed for 21 days, i.p., in the presence and absence of rosmarinic acid (0.2805 mg/kg/day (0.05 LD_50_), 21 days, i.g.) or carvacrol (0.0405 mg/kg/day (0.05 LD_50_), 21 days, i.g.). The treatment with AlCl_3_ increased GSH concentration in erythrocytes only slightly and had no effect on brain and liver homogenates. Rosmarinic acid and carvacrol strongly increased GSH concentration in erythrocytes but decreased it in brain and liver homogenates. However, AlCl_3_ treatment led to Al accumulation in mice blood, brain, and liver and induced oxidative stress, assessed based on MDA concentration in the brain and liver. Both rosmarinic acid and carvacrol were able to counteract the negative Al effect by decreasing its accumulation and protecting tissues from lipid peroxidation. AlCl_3_ treatment increased CAT activity in mice brain and liver homogenates, whereas the administration of either rosmarinic acid or carvacrol alone or in combination with AlCl_3_ had no significant effect on CAT activity. SOD activity remained unchanged after all the treatments in our study. We propose that natural herbal phenolic compounds rosmarinic acid and carvacrol could be used to protect brain and liver against aluminum induced oxidative stress leading to lipid peroxidation.

## 1. Introduction

Aluminum (Al) is the third most abundant chemical element found in nature and the most abundant metal in the Earth′s crust [1]. It is widely used in the medical, food, and pharmaceutical industries as a part of vaccines, for water purification, for packaging, and in agriculture, thus resulting in human exposure [2]. The toxicity of different Al forms depends on their physical behavior and relative solubility in water [1]. After delivery to target tissues, elevated Al^3+^ concentrations induce cytotoxicity which is a consequence of oxidative damage due to Al^3+^-induced formation of oxygen radicals [1,3,4,5,6,7]. Aluminum can cause nephrotoxicity [8], cardiotoxicity [9,10], hematotoxicity [11], hepatotoxicity [12], and bone and lung toxicity [9,10]. Aluminum easily crosses the blood–brain barrier and can accumulate in different regions of the brain [13,14], inducing neurotoxicity and contributing to neuronal death in neurodegenerative diseases such as Alzheimer′s disease and Parkinson′s disease [15,16,17]. Due to frequent human exposure to Al and the possible role of Al in neurodegenerative disorders, it is important to find substances that could potentially counteract the toxic effects.

There is accumulating evidence that natural herbal remedies and dietary compounds could have potential therapeutic use in the treatment of numerous neurodegenerative diseases [18]. Several studies have demonstrated that polyphenols and endogenous compounds could alleviate neuronal oxidative damage and inflammation [19,20] and counteract metabolic disorders associated with these diseases [21,22]. Rosmarinic acid is the ester of caffeic acid and 3,4-dihydroxyphenyl-lactic acid and one of the most abundant phenols in many well-known herbs of the *Lamiaceae* family [23], such as rosemary, sage, basil, mint, and thyme. Among the simple phenolic compounds, rosmarinic acid has been shown to possess very strong antioxidant activity [24]. Rosmarinic acid was demonstrated to have neuroprotective properties, attenuate oxidative stress and neuronal cell death in vitro [25,26,27], and to diminish inflammatory responses in experimental models of an ischemic stroke [18,28]. Carvacrol is a monoterpenoid phenol and a major constituent of oregano oil, which is widely used as a salad dressing, and numerous other essential oils [29]. Carvacrol exhibits strong antioxidative and hydrophobic properties associated with the substituted aromatic ring and hydrophilic properties associated with the phenolic OH group [29,30]. It has been demonstrated to exert antioxidative, anti-inflammatory, antibacterial, antiviral, antifungal, antiprotozoal, anticarcinogenic, antidiabetic, antinociceptive, cardioprotective, and neuroprotective activities [31,32,33]. Rosmarinic acid and carvacrol could be readily available potential remedies to counteract the toxic effects of Al. Therefore, in this study, we investigated the effects of rosmarinic acid and carvacrol on oxidative stress and antioxidant enzyme activity in Al-treated mice.

## 2. Results

We used in our study experimental setup where BALB/c mice were treated intraperitoneally with Al^3+^ 7.5 mg/kg/day [0.15 LD_50_] for 21 days, which is considered a very low dose of Al. During such exposure, we have not observed neither mice behavioral changes nor the changes at histological level. The main purpose of our study was to assess the first possible toxicological effects of low dose Al^3+^ exposure that usually occur at subcellular levels.

### 2.1. Cl^−^ Controls

AlCl_3_ was used as the source of Al^3+^ in the study. To determine whether the effects observed are related to the Cl^−^, equimolar Cl^−^ controls were performed for all endpoints studied, and the results showed no difference between control and HCl treatments.

### 2.2. Effects of AlCl_3_, Rosmarinic Acid, and Carvacrol Treatment on the Amount of Al in Mice Blood and Brain and Liver Tissues 

**Figure 1:**
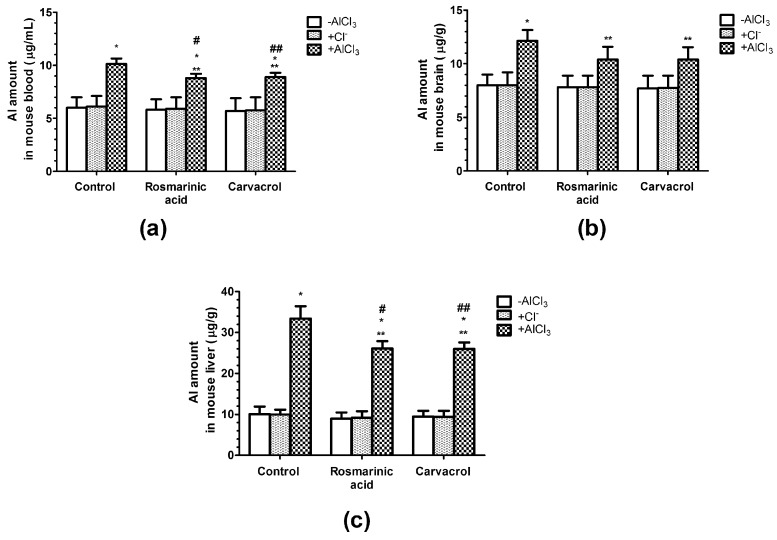
Influence of AlCl_3_, rosmarinc acid, and carvacrol on the amount of Al in mice blood (**a**) and brain (**b**) or liver (**c**) tissues. (*n* = 9, * *p* < 0.001 vs. control, ** *p* < 0.001 vs. AlCl_3_, # *p* < 0.01 vs. rosmarinic acid in the absence of AlCl_3_, ## *p* < 0.01 vs. carvacrol in the absence of AlCl_3_).

The amount of Al in mice blood was significantly increased by 69% compared to the vehicle control (6 µg/mL) in the case of AlCl_3_, whereas the administration of rosmarinic acid or carvacrol in the presence of AlCl_3_ decreased its effect by 30% (Figure 1a).

After AlCl_3_ treatment, the amount of Al in mice brain and liver (Figure 1b,c) was significantly increased by 52.2% and 230% accordingly, compared to the vehicle control (8 µg/g in brain and 10 µg/g in liver). The administration of rosmarinic acid or carvacrol in the presence of AlCl_3_ decreased its effect by 40% in brain and by 30% in liver. (Figure 1b,c).

### 2.3. Effects of AlCl_3_, Rosmarinic Acid, and Carvacrol Treatment on the Concentration of Gsh in Mice Erythrocytes and Brain and Liver Homogenates

**Figure 2:**
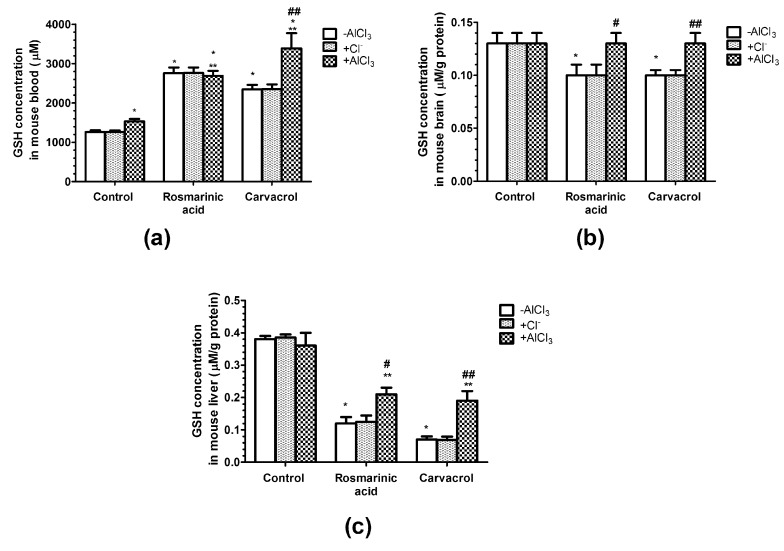
Influence of aluminum ions, rosmarinc acid, and carvacrol on the concentration of glutathione (GSH) in mice erythrocytes (**a**) and brain (**b**) or liver (**c**) homogenates. (*n* = 9, * *p* < 0.001 vs. control, ** *p* < 0.001 vs. AlCl_3_, # *p* < 0.01 vs. rosmarinic acid in the absence of AlCl_3_, ## *p* < 0.01 vs. carvacrol in the absence of AlCl_3_).

The concentration of the intracellular antioxidant GSH in mice erythrocytes was significantly increased by 22% in the case of AlCl_3_, by 219% in the case of rosmarinic acid, and by 87% in the case of carvacrol administration compared with the vehicle control (Figure 2a). In mice treated with both AlCl_3_ and rosmarinic acid, the significant increase in erythrocyte GSH concentration remained similar to that in the case of rosmarinic acid administration alone (214%). In mice treated with both AlCl_3_ and carvacrol, the increase in erythrocyte GSH concentration was significantly augmented by 270% compared with the vehicle control (Figure 2a).

However, the different effects of AlCl_3_, rosmarinic acid, and carvacrol on GSH concentration was observed in mice brain and liver homogenates (Figure 2b,c). Rosmarinic acid and carvacrol treatment significantly decreased the GSH concentration in mice brain homogenates by 30%, whereas AlCl_3_ treatment alone or in combination with rosmarinic acid or carvacrol had no effect compared with the vehicle control (Figure 2b). AlCl_3_ treatment had no effect on GSH concentration in mice liver homogenates, whereas rosmarinic acid administration significantly decreased it by 68% alone or by 45% together with AlCl_3_ compared with the vehicle control (Figure 2c). A similar pattern was observed in the case of carvacrol administration—it significantly decreased the GSH concentration in mice liver homogenates by 92% alone or by 50% in combination with AlCl_3_ compared with the vehicle control (Figure 2c).

### 2.4. Effects of AlCl_3_, Rosmarinic Acid, and Carvacrol Treatment on the Concentration of MDA in Mice Erythrocytes and Brain and Liver Homogenates

**Figure 3:**
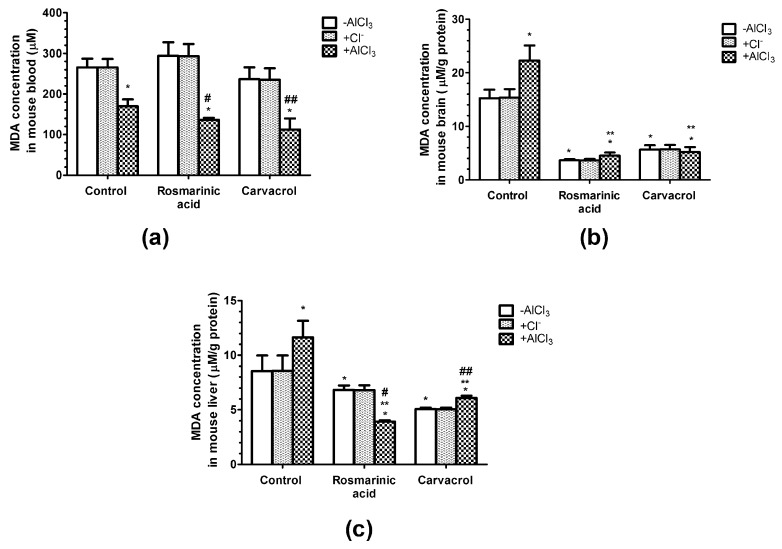
Influence of aluminum ions, rosmarinc acid, and carvacrol on the concentration of malondialdehyde (MDA) in mice erythrocytes (**a**) and brain (**b**) or liver (**c**) homogenates. (*n* = 9, * *p* < 0.001 vs. control, ** *p* < 0.001 vs. AlCl_3_, # *p* < 0.001 vs. rosmarinic acid in the absence of AlCl_3_, ## *p* < 0.001 vs. carvacrol in the absence of AlCl_3_).

The concentration of the final product of lipid peroxidation (MDA) was significantly decreased by 36%, 48%, and 67% in mice erythrocytes treated with AlCl_3_ alone or in combination with rosmarinic acid and carvacrol, respectively, compared with the vehicle control (Figure 3a).

In mice brain homogenates, AlCl_3_ administration significantly increased the concentration of MDA by 46%, whereas rosmarinic acid administration alone or in combination with AlCl_3_ significantly decreased it by 76% and 70%, respectively, compared with the vehicle control (Figure 3b). Similarly, mice treated with carvacrol alone or in combination with AlCl_3_ showed significant decreases in MDA concentration by 63% and 66%, respectively (Figure 3b). Comparable effects of AlCl_3_, rosmarinic acid, and carvacrol treatments were observed in mice liver homogenates (Figure 3c). AlCl_3_ significantly increased the MDA concentration by 36%, whereas rosmarinic acid and carvacrol significantly decreased it by 20% and 41%, respectively, when administered alone and by 54% and 29%, respectively, in combination with AlCl_3_ (Figure 3c).

### 2.5. Effects of AlCl_3_, Rosmarinic Acid, and Carvacrol Treatment on the Activities of CAT and SOD in Mice Brain and Liver Homogenates

**Figure 4:**
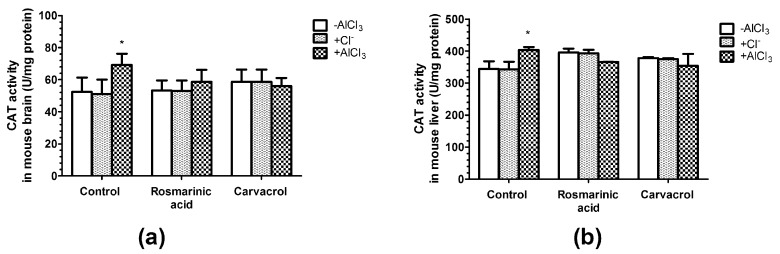
Influence of aluminum ions, rosmarinc acid, and carvacrol on the activity of catalase (CAT) in mice brain (**a**) and liver (**b**) homogenates (*n* = 9, * *p* < 0.05 vs. control).

CAT activity was observed to be 32% higher (*p* < 0.05) in brain homogenates (Figure 4a) and 17% higher (*p* < 0.05) in liver homogenates (Figure 4b) among AlCl_3_-treated mice.

**Figure 5:**
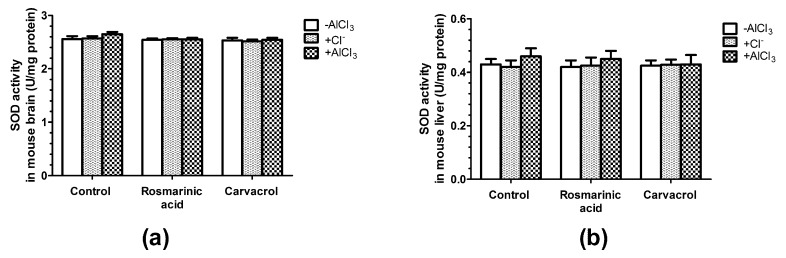
Influence of aluminum ions, rosmarinc acid, and carvacrol on the activity of superoxide dismutase (SOD) in mice brain (**a**) and liver (**b**) homogenates (*n* = 9).

The administration of rosmarinic acid and carvacrol alone or in combination with AlCl_3_ had no significant effects on CAT activity (Figure 4a,b). Changes in SOD activity followed the same pattern; no effects were observed in the case of all treatments except with negligible changes noticed in mice treated with AlCl_3_ alone (Figure 5a,b).

## 3. Discussion

Numerous studies have demonstrated the toxicity of aluminum in various animal models [1]. The most important concern about aluminum exposure is related to its neurotoxic effects and its possible role in neurodegenerative disorders [6,34]. Most literature data about the low dose Al toxicity is based on the experiments where Al is administered orally. Direct evidence of neurotoxicity due to low doses of Al is controversial, e.g., Shoji et al. reported that 0.97–9.7 mg/kg/day Al per os for 60 days did not induce statistically significant behavioral changes in C57BL/6J mice [35], whereas Martinez et al. found that 8.3 mg/kg/day Al per os for 60 days promoted the development of mechanical allodynia, catalepsy, increased inflammation in the sciatic nerve, and systemic oxidative stress, and was able to be retained in the sciatic nerve [36].

The toxicity of soluble Al forms depends upon the delivered dose of Al^3+^ to target tissues. Elevated Al^3+^ concentrations lead to Al^3+^—induced formation of oxygen radicals causing lipid peroxidation, which results in cell membrane damage and oxidative stress [3,4,5,6,7]. Accumulating evidence obtained from in vitro, in vivo, and clinical studies supports the free radical scavenging properties of natural phenolic antioxidants [24]. In this study, we proposed that natural herbal phenolic compounds with strong antioxidant activity, such as rosmarinic acid [24] and carvacrol [29], could help to counteract the toxicity of Al.

Endogenous ROS are mainly produced in the mitochondrial inner membrane during the process of oxidative phosphorylation. The uncontrolled formation of free radicals and activated oxygen species is prevented by intracellular antioxidant systems [37]. Intracellular antioxidants include low molecular weight scavengers of oxidizing species (such as GSH), and enzymes that degrade superoxide and hydroperoxides [37,38]. GSH acts as the main non-enzymatic antioxidant that directly scavenges free radicals or serves as a substrate for the glutathione peroxidase, the enzyme that catalyzes the detoxification of H_2_O_2_ [37,39]. MDA is the end product of lipid peroxidation, and its level indicates the extent of oxidative stress [40,41,42].

We assessed Al amount, GSH and MDA concentrations as well as CAT and SOD activities in erythrocytes and brain and liver homogenates of BALB/c mice treated with Al^3+^ (7.5 mg/kg/day (0.15 LD_50_), 21 days, i.p.) in the presence and absence of rosmarinic acid (0.2805 mg/kg/day (0.05 LD_50_), 21 days, i.g.) or carvacrol (0.0405 mg/kg/day (0.05 LD_50_), 21 days, i.g.).

The Al amount in mice blood, brain and liver tissues increased after AlCl_3_ treatment by 69%, 52.2%, and 230% accordingly, compared to control values (Figure 1a–c). Rosmarinic acid or carvacrol diminished the accumulation of Al by 30% in blood, by 40% in brain, and by 30% in liver tissues (Figure 1a–c). Thus, the highest degree of protection from Al accumulation by rosmarinic acid or carvacrol was observed in brain in our study.

Our results showed that the concentration of intracellular antioxidant GSH in erythrocytes in mice treated with rosmarinic acid or carvacrol in either the presence or absence of Al^3+^ increased (*p* < 0.05; Figure 2a). This effect was even greater in the case of both carvacrol and Al. However, in brain (Figure 2b) and liver (Figure 2c) homogenates, the GSH concentration was not affected by Al^3+^ treatment but was diminished by rosmarinic acid and carvacrol (*p* < 0.05). Some studies have shown that Al^3+^ reduces the brain GSH contents in Sprague-Dawley rats (10 mg/kg/day, 45 days, i.p.) [43]. Viezeliene et al. also found that in BALB/c mice treated with Al^3+^ i.p. once (25 mg/kg, sample taken after 16 h), liver GSH activity was significantly decreased compared with normal controls [12]. However, other studies have indicated that Al^3+^ increases GSH levels in all brain regions of Swiss albino mice (50 mg/kg/d, 42 days, per os (p.o.)) [44]. These inconsistencies may be due to differences among animals, dosages, times, experimental conditions, or experimental procedures [45,46]. Thus, in our study, treatment with Al^3+^ (7.5 mg/kg/day [0.15 LD_50_], 21 days, i.p.) led to only a slight increase in GSH concentration in erythrocytes and had no significant effects on brain and liver homogenates. Rosmarinic acid promotes an increase in intracellular GSH concentrations [47]. However, the opposite effect—a decrease in GSH caused by flavonoids—has been reported in several studies [48,49]. In the case of a phenol ring containing dietary polyphenolics treatment, some GSH was oxidized to glutathione disulfide [49]. It has also been reported that both antioxidant and pro-oxidant activities could be observed at different doses of phenolic compounds [50]. Thus, the strongly increased concentration of the first-line non-enzymatic radical scavenger GSH in erythrocytes in our study (Figure 2a) could be related to the antioxidant properties of rosmarinic acid and carvacrol, and the decrease in GSH content (although to a lesser extent) in brain (Figure 2b) and liver (Figure 2c) to their pro-oxidant activities.

MDA is the marker of lipid peroxidation, its increase is related to the negative effects on the fluidity and damage to cell membranes, and it is implicated as the most important parameter that shows the extent of oxidative stress [42]. In our study, MDA levels were decreased (*p* < 0.05) in the erythrocytes of mice that received Al^3+^ alone or in combination with rosmarinic acid and carvacrol (Figure 3a). However, MDA concentration significantly increased after Al^3+^ administration, whereas rosmarinic acid and carvacrol treatment alone or in combination with Al^3+^ resulted in highly decreased MDA levels in mice brain (Figure 3b) and liver (Figure 3c) homogenates. Our results indicate that AlCl_3_ induced oxidative stress in the brain and liver, and both rosmarinic acid and carvacrol were able to counteract the negative Al effect and protect against lipid peroxidation. The toxic effect of Al could be related to its accumulation in brain and liver to a higher extent than in the erythrocytes in the blood. This is in good agreement with observations showing that Al is mainly absorbed by the gastrointestinal tract and easily accumulates in body tissues [51,52], thus causing central nervous system toxicity, hepatotoxicity, nephrotoxicity, cardiotoxicity, and osteoporosis [53,54,55]. Rosmarinic acid has been shown to be neuroprotective, attenuating oxidative stress and neuronal cell death in vitro [25,26,27] and reducing inflammatory responses in experimental models of an ischemic stroke [18,28]. Rosmarinic acid treatment decreases MDA in rat liver [56], as well as in mice brain [57], kidney, and liver [57,58]. Carvacrol decreases the peroxidation of phospholipid liposomes in the presence of iron(III) and ascorbate [59], and inhibits low-density lipoprotein oxidation in a concentration dependent manner in an in vitro system using human aortic endothelial cells [60]. Reduced levels of MDA have also been found in homogenates of carvacrol-treated rat hearts [61,62]. The ability of rosmarinic acid and carvacrol to significantly diminish the levels of MDA in mice brain (Figure 3b) and liver (Figure 3c) in our study suggests the potential of these natural phenolic compounds to act as brain and liver protectants via the antioxidant effect.

SOD and CAT are considered primary enzymes of the antioxidant system, which is involved in the direct elimination of ROS. SOD converts superoxide anion to hydrogen peroxide and oxygen, and diminishes the toxic effects due to this radical or other free radicals derived from secondary reactions [37,38]. CAT is a hemoprotein that catalyzes the conversion of hydrogen to H_2_O [38]. Our results have shown that only AlCl_3_ treatment increased CAT activity (*p* < 0.05) in mice brain and liver homogenates (Figure 4a,b), whereas rosmarinic acid or carvacrol administration alone or in combination with Al^3+^ had no significant effects on CAT activity (Figure 4a,b). SOD activity remained unchanged after all treatments in our study (Figure 5a,b). The data on Al^3+^ influence on the activities of antioxidant enzymes is not consistent, and might depend on the dosage, treatment duration, and experimental conditions [45,46]. Al^3+^ was shown to decrease brain CAT activity in Swiss albino mice (50 mg/kg/day, 60 days, p.o.) [63], and decrease SOD activity in specific brain regions of Swiss albino mice (50 mg/kg/day, 42 days, p.o.) [44]. However, other studies have shown that Al^3+^ increases SOD activity in the brain in Swiss albino mice (100 mg/kg/day, 42 days, i.p.) [64]. Moreover, one report showed that brain CAT activity significantly decreased, whereas brain SOD activity was not altered in Al^3+^ treated Kunming mice (40 mg/kg/day, i.p., 28 days) [46]. The impact of rosmarinic acid and carvacrol on antioxidant enzyme activity also depends on their dosage. In our case, to avoid toxic effects and to mimic dietary consumption of these compounds, we used rosmarinic acid and carvacrol at doses corresponding to 0.05 LD_50_ (0.2805 mg/kg/day and 0.0405 mg/kg/day, respectively). Under these conditions, both rosmarinic acid and carvacrol were able to prevent lipid peroxidation by Al and to keep MDA at low levels (Figure 3). However, no significant effects of rosmarinic acid or carvacrol on brain and liver CAT (Figure 4) and SOD (Figure 5) activities were observed. Other studies have reported that CAT and SOD are activated by rosmarinic acid [65] and carvacrol [62]. However, much higher phenolic compound doses were administered in these investigations, at levels of up to 100 times higher than we used in our study.

## 4. Materials and Methods

### 4.1. Materials

Rosmarinic acid (>98%) was purchased from ChromaDex (Santa Ana, TX, USA). All other chemicals, including carvacrol (>98%) and AlCl_3_ (>99.9%), were from Sigma-Aldrich (St. Louis, MO, USA).

### 4.2. Animal Model

The experiments were performed on 4 to 6-week-old white male BALB/c laboratory mice weighing 20–25 g that were housed at 23 ± 2 °C with a 12 h light/dark cycle and free access to food and water. The mice were from the Vivarium of Lithuanian University of Health Sciences and were kept there during the experiments. According to the requirements of the EU Directive 2010/63/EU for animal experiments the study protocol was approved by the Lithuanian State Food and Veterinary Service, License No. G2–19. Mice were randomly allocated to nine groups (*n* = 9/group) kept in separate cages: vehicle control, AlCl_3_, rosmarinic acid, rosmarinic acid + AlCl_3_, carvacrol, carvacrol + AlCl_3_, and three Cl^−^ control groups (HCl, rosmarinic acid + HCl, and carvacrol + HCl). AlCl_3_ dissolved in saline was injected intraperitoneally (i.p.) at a dose of 7.5 mg of Al^3+^/kg body weight (0.15 LD_50_) in the AlCl_3_, rosmarinic acid + AlCl_3_, and carvacrol + AlCl_3_ mouse groups for 21 days. In the Cl^−^ control groups (HCl, rosmarinic acid + HCl, and carvacrol + HCl), mice received an equimolar to AlCl_3_ dose of Cl^−^ as HCl (30.41 mg/kg/day for 21 days, i.p.). Mice in the vehicle control, rosmarinic acid, and carvacrol groups received an equal volume of saline in the same manner, mice in the rosmarinic acid, rosmarinic acid + HCl, and rosmarinic acid + AlCl_3_ groups received intragastric (i.g.) rosmarinic acid (0.2805 mg/kg body weight, corresponding to 0.05 LD_50_) for 21 days, and the mice in the carvacrol, carvacrol + HCl, and carvacrol + AlCl_3_ groups received carvacrol (0.0405 mg/kg body weight, corresponding to 0.05 LD_50_) for 21 days, all diluted in saline. To avoid toxic effects and to mimic dietary consumption of these compounds, we used rosmarinic acid and carvacrol at doses corresponding to 0.05 LD_50_ (0.2805 mg/kg/day and 0.0405 mg/kg/day, respectively). AlCl_3_ was used i.p. at a dose of 7.5 mg of Al^3+^/kg body weight (0.15 LD_50_) to avoid animal deaths due to excess accumulation of Al in prolonged experiment and to mimic low doses of Al found in vaccines. 81 mice were used in total.

### 4.3. Sample Preparation

After the exposure time, the blood samples were collected in the pre-heparinized tubes. The blood was centrifuged for 10 min at 800× *g* at 4 °C, and the plasma layers with debris were removed by aspiration. The erythrocytes were washed three times with a phosphate-buffered saline (pH 7.4) and kept on ice.

The animals were terminated according to the rules defined by the EU Directive 2010/63/EU for animal experiments. Following that, the brain and liver were quickly removed, put on Petri dishes, and immediately cooled in an ice bath. Homogenates were prepared as described below. Protein concentrations in the brain and liver homogenate samples were determined by the Warburg-Christian method.

The erythrocytes and homogenate samples were coded for the endpoint measurements. The assessors have not got the information which sample belongs for which experimental group.

### 4.4. Determination of Al Amount in Blood and Target Tissues

The concentration of Al in mouse erythrocytes, liver and brain was evaluated by using an inductively coupled plasma mass spectrometer NexION 300 D (PerkinElmer, Inc. Shelton, CT, USA ). Tissue specimens were digested with 0.125 M NaOH at 90 °C. The digests were diluted to the appropriate volume and were analyzed according to the manufacturer′s recommendations for the detection of Al concentrations in biological samples. To ensure the accuracy of the analysis, we conducted internal and external quality control procedures, including the use of analytical high-purity water and reagents (Sigma-Aldrich Chemie GmbH, Taufkirchen, Germany), certified reference materials ClinCheck^®^ Whole Blood Controls Level (Recipe Chemicals + Instruments GmbH, München, Germany), Standard Reference Material^®^−1577c bovine liver. We also conducted the control of labware for contamination with Al.

### 4.5. Determination of Glutathione Level

The amount of glutathione (GSH), an intracellular antioxidant, was assessed based on a reduced GSH reaction with the 5,5′-dithiobis-(2-nitrobenzoic acid) (DTNB) in an alkaline environment, which produces a yellow complex.

GSH concentration in erythrocytes was determined according to the method of Sedlak and Lindsay [66]. Erythrocytes (200 µL) were added to 1.8 mL of deionized water and 2 mL of 0.6 M HClO_4_. The mixture was centrifuged for 10 min at 3000× *g* and the upper aqueous layer was decanted to the test tube and used for the color reaction. Supernatant (1 mL) was added to 3 mL of 0.4 M Tris-HCl (pH 9.2) and 50 µL of DTNB stock solution (3.7 mg of DTNB + 1 mL ethanol). Light absorbance of the solution was determined spectrophotometrically at 412 nm.

GSH concentration in liver and brain homogenates was determined according to the method of Moron et al. [67]. Liver and brain homogenates were prepared with 6 volumes (*w*/*v*) of 5% trichloroacetic acid (TCA) solution. The homogenates were centrifuged at 10,000× *g* for 7 min to obtain GSH-containing supernatants. The supernatant (0.2 mL) was mixed with 2 mL of 0.6 mM DTNB in 0.2 M phosphate buffer (pH 8.0), and 0.8 mL of phosphate buffer was added to make a final reaction volume of 3 mL. The light absorbance of the solution was determined spectrophotometrically at 412 nm. A mixture of buffered DTNB solution containing 0.2 mL of 5% TCA was used as a reference. GSH concentration was expressed as µM/g protein or µM (in case of erythrocytes).

### 4.6. Determination of Malondialdehyde Level

The amount of malondialdehyde (MDA), a marker of lipid peroxidation, was estimated in erythrocytes, liver, and brain homogenates by measuring thiobarbituric acid reactive substances.

Thiobarbituric acid (2 mL of 0.5%) was added to the reaction mixture containing 2 mL of deionized water, 0.1 mL of erythrocytes, and 1 mL of 10% TCA solution. The mixture was heated for 30 min in a boiling water bath. After cooling in the ice bath, the mixture was centrifuged for 15 min at 3000× *g* at 4 °C. The supernatant was filtered through four layers of sterile gauze and the light absorbance was determined spectrophotometrically at 540 nm [68].

Liver and brain homogenates were prepared with nine volumes (*w*/*v*) of cold 1.15% KCl. H_3_PO_4_ (3 mL of 1%) and thiobarbituric acid (1 mL of 0.6%) aqueous solutions were added to 0.5 mL of the homogenates. The mixtures were heated for 45 min in a boiling water bath. After cooling, 4 mL of *n*-butanol was added and the solution was mixed vigorously. The butanol phase was separated by centrifugation. The light absorbance of the supernatants was then determined spectrophotometrically at 535 and 520 nm [69]. MDA concentrations were expressed as µM (in case of erythrocytes) or µM/g protein.

### 4.7. Catalase Activity Assay

The activity of catalase (CAT) in mice brain and liver homogenates was determined by the hydrogen peroxide reaction with ammonium molybdate, which produces a complex that absorbs light at 410 nm [70]. Enzyme activity units were equal to the amount of enzyme that consumes 1 µM H_2_O_2_ within a minute under the experimental conditions, and were reported as U/mg protein.

### 4.8. Superoxide Dismutase Activity Assay

The activity of superoxide dismutase (SOD) in mice brain and liver homogenates was determined according to the inhibition of nitroblue tetrazolium reduction rate in the non-enzymatic phenazine methosulfate-nicotinamide adenine dinucleotide system assessed spectrophotometrically at 540 nm [70]. The SOD activity was expressed as U/mg protein, where U was the relative unit of activity defined as the amount of SOD required for the inhibition of nitroblue tetrazolium reduction by 50%.

### 4.9. Statistics

Data are expressed as the mean ± standard error of the mean (*n* = 9). Statistical analysis was performed by one-way analysis of variance, followed by Tukey′s post-test using the Prism v. 5.04 software package (GraphPad Software Inc., La Jolla, CA, USA). A value of *p* < 0.05 was taken as the level of significance.

## 5. Conclusions

To conclude, we have shown that prolonged exposure to Al^3+^, even at considerably low doses (0.15 LD_50_), resulted in Al accumulation and induced oxidative stress in mice brain and liver, and rosmarinic acid and carvacrol were able to counteract the negative Al effect decreasing Al accumulation and protecting tissues from lipid peroxidation. Since aluminum-induced toxicity has been suggested to have a role in the development of neurodegenerative disorders, the natural herbal phenolic compounds rosmarinic acid and carvacrol widely used as foods or food additives in the food industry could be important candidates in the attenuation of oxidative stress and provide protection from related injuries.
